# A structural view of synthetic cofactor integration into [FeFe]-hydrogenases†
†Electronic supplementary information (ESI) available: Tables listing and comparing the RMSD of the structures, distances and angles of the 2Fe_H_-subclusters, the distances from 2Fe_H_-subcluster atoms to selected amino acids and the distances of amino acids lining the 2Fe_H_-subcluster cavity, a figure showing the presumed maturation channel in more detail and additional information on the suggested glycine hinges and stereo views of all figures presented in the main article. See DOI: 10.1039/c5sc03397g


**DOI:** 10.1039/c5sc03397g

**Published:** 2015-10-26

**Authors:** J. Esselborn, N. Muraki, K. Klein, V. Engelbrecht, N. Metzler-Nolte, U.-P. Apfel, E. Hofmann, G. Kurisu, T. Happe

**Affiliations:** a AG Photobiotechnologie , Fakultät für Biologie und Biotechnologie , Ruhr-Universität Bochum , Universitätsstraße 150 , 44801 Bochum , Germany . Email: thomas.happe@rub.de; b Laboratory of Protein Crystallography , Institute for Protein Research , Osaka University , Suita , Osaka 565-0871 , Japan . Email: gkurisu@protein.Osaka-u.ac; c Lehrstuhl für Anorganische Chemie I—Bioanorganische Chemie , Fakultät für Chemie und Biochemie , Ruhr-Universität Bochum , Universitätsstraße 150 , 44801 Bochum , Germany . Email: ulf.apfel@rub.de; d AG Proteinkristallographie , Fakultät für Biologie und Biotechnologie , Ruhr-Universität Bochum , Universitätsstraße 150 , 44801 Bochum , Germany

## Abstract

[FeFe]-hydrogenases are nature's fastest catalysts for the evolution or oxidation of hydrogen. Numerous synthetic model complexes for the [2Fe] subcluster (2Fe_H_) of their active site are known, but so far none of these could compete with the enzymes. The complex Fe_2_[μ-(SCH_2_)_2_X](CN)_2_(CO)_4_^2–^ with X = NH was shown to integrate into the apo-form of [FeFe]-hydrogenases to yield a fully active enzyme. Here we report the first crystal structures of the apo-form of the bacterial [FeFe]-hydrogenase CpI from *Clostridium pasteurianum* at 1.60 Å and the active semisynthetic enzyme, CpI^ADT^, at 1.63 Å. The structures illustrate the significant changes in ligand coordination upon integration and activation of the [2Fe] complex. These changes are induced by a rigid 2Fe_H_ cavity as revealed by the structure of apoCpI, which is remarkably similar to CpI^ADT^. Additionally we present the high resolution crystal structures of the semisynthetic bacterial [FeFe]-hydrogenases CpI^PDT^ (X = CH_2_), CpI^ODT^ (X = O) and CpI^SDT^ (X = S) with changes in the headgroup of the dithiolate bridge in the 2Fe_H_ cofactor. The structures of these inactive enzymes demonstrate that the 2Fe_H_-subcluster and its protein environment remain largely unchanged when compared to the active enzyme CpI^ADT^. As the active site shows an open coordination site in all structures, the absence of catalytic activity is probably not caused by steric obstruction. This demonstrates that the chemical properties of the dithiolate bridge are essential for enzyme activity.

## Abstract 2

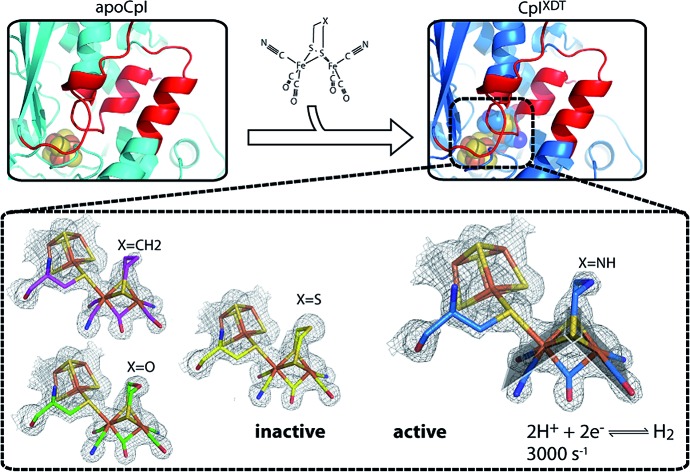
Crystal structures of semisynthetic [FeFe]-hydrogenases with variations in the [2Fe] cluster show little structural differences despite strong effects on activity.

## Introduction

[FeFe]-hydrogenases are efficient natural catalysts for both the generation and oxidation of H_2_.^1^ This reaction is accomplished by the H-cluster, a metal cofactor consisting of a cubane [4Fe4S] cluster (4Fe_H_) connected *via* a cysteine to an unusual [2Fe] cluster (2Fe_H_). The two iron atoms of the latter, termed distal (Fe_d_) and proximal (Fe_p_) iron are ligated by a total of three CO and two CN^–^ molecules and an aza-dithiolato bridge.^2^–^5^ Four structural characteristics seem to be important for the high activity of the H-cluster:^6^,^7^ (a) The cyanide ligands besides having additional effects^8^–^10^ shift the redox potential to more negative values, when compared to all–carbonyl complexes.^11^ (b) The 4Fe_H_-cluster serves as an intramolecular redox partner.^12^–^15^ (c) A proton donor is present in the dithiolato bridge.^16^–^19^ (d) The ligand conformation at the 2Fe_H_ subsite features a CO that bridges or semi-bridges the Fe atoms. This leads to an unoccupied coordination site on Fe_d_.^3^,^5^,^20^–^22^ For the hydrogenase HydA1 from *Chlamydomonas reinhardtii* three redox states are discussed as part of the catalytic cycle, which can be distinguished by EPR and FTIR spectroscopy. According to this hypothesis the H-cluster cycles from the H_ox_ state 4Fe_H_^2+^–Fe(i)–Fe(ii), *via* the H_red_ state 4Fe_H_^2+^–Fe(i)–Fe(i), to the H_sred_ state 4Fe_H_^1+^–Fe(i)–Fe(i). The redox potentials for these transitions are –400 mV and –470 mV *vs.* SHE close to the H_2_/H^+^ redox pair.^7^ The crucial proton transfer to and from the active site seems to be accomplished by a proton transfer pathway through the protein towards the central atom of the dithiolato bridge in the 2Fe_H_-subcluster.^3^,^5^,^23^

In nature, the 4Fe_H_-cluster and other FeS clusters of the enzyme not specific to [FeFe]-hydrogenases are synthesized by the widespread ISC or SUF systems for FeS cluster synthesis yielding inactive hydrogenases, which lack only the specific 2Fe_H_-subcluster.^24^ For the sake of simplicity this pre-form will be referred to as apo-form of [FeFe]-hydrogenases throughout this text. The three maturase enzymes HydE, HydF and HydG are necessary for the synthesis of the 2Fe_H_-cluster and the assembly of the H-cluster within the protein.^25^*In vitro*, chemically synthesized [2Fe] complexes can be bound to the maturase HydF and transferred from there to apo-hydrogenases to form a complete H-cluster.^2^ Notably, also in the absence of HydF or any other helper protein, an active H-cluster can be formed spontaneously by bringing together the inactive apo-hydrogenase and the chemically synthesized [2Fe] complex Fe_2_[μ-(SCH_2_)_2_NH](CN)_2_(CO)_4_^2–^.^26^ While the [2Fe] moiety alone is inactive under physiological conditions, the semisynthetic enzyme shows high catalytic activity, which demonstrates the importance of the protein environment. [2Fe] complexes with variations in the dithiolato bridge and/or the other Fe ligands have recently been shown to integrate into HydA1 as well, but the enzymes were inactive or severely limited in their turnover rates especially if the dithiolato bridge was changed.^2^,^27^ The central atom of the dithiolato-moiety seems to influence the redox behavior of the H-cluster either directly or by interfering with the proton transfer to/from the active site.^28^

Structures of the active bacterial [FeFe]-hydrogenases^3^,^29^,^30^ and the inactive apo-form of HydA1 from *Chlamydomonas reinhardtii*^31^ have been solved at high resolution. In this study we aim to expand the knowledge about maturation of [FeFe]-hydrogenases by reporting the crystal structure of the [FeFe]-hydrogenase from *Clostridium pasteurianum* (CpI) in its apo-form without the 2Fe_H_-cluster (apoCpI). In addition, we contribute to a deeper understanding of [FeFe]-hydrogenase function by solving the structures of four semisynthetic hydrogenases maturated *in vitro* with [2Fe] complexes of the kind Fe_2_[μ-(SCH_2_)_2_X](CN)_2_(CO)_4_^2–^: fully active CpI^ADT^ (X = NH) and its non-active derivatives CpI^PDT^ (X = CH_2_), CpI^ODT^ (X = O) and CpI^SDT^ (X = S).

## Results and discussion

### Only an ADT-bridged [2Fe] cluster induces H_2_ evolution activity in CpI

To compare the structure of active semisynthetic CpI containing the ADT-bridged 2Fe_H_-subcluster with the native bacterial hydrogenase and to investigate potential structural aspects of the impaired function of the semisynthetic enzyme derivatives with different dithiolato bridges, apoCpI was maturated with four synthetic [2Fe] clusters. Besides the ADT-bridged [2Fe] complex (Fe_2_[μ-(SCH_2_)_2_NH](CN)_2_(CO)_4_^2–^), a PDT-bridged [2Fe] complex (Fe_2_[μ-(SCH_2_)_2_CH_2_](CN)_2_(CO)_4_^2–^), an ODT-bridged [2Fe] complex (Fe_2_[μ-(SCH_2_)_2_O](CN)_2_(CO)_4_^2–^) and an SDT-bridged [2Fe] complex (Fe_2_[μ-(SCH_2_)_2_S](CN)_2_(CO)_4_^2–^) were synthesized following modified literature procedures^32^–^38^ and used to prepare semisynthetic CpI as described before.^26^ Specific hydrogen evolution activities with methylviologen as electron donor were 2874 ± 262 (μmol H_2_) min^–1^ (mg protein)^–1^ for CpI with the ADT-bridged 2Fe_H_-cluster (CpI^ADT^), which is in agreement with previously reported values.^10^,^26^ Neither for apoCpI nor for the non-natural derivatives CpI^PDT^, CpI^ODT^ or CpI^SDT^ could any hydrogen evolution activity be detected above the detection limit of 0.02% of the activity of CpI^ADT^. The same [2Fe] complexes were recently integrated into HydA1. While the ODT-bridged and SDT-bridged complexes didn't induce H_2_ evolution, 0.9 (μmol H_2_) min^–1^ (mg protein)^–1^ were reportedly produced by HydA1 with the PDT-bridged 2Fe_H_-cluster. This equals 0.17% of the activity of the same enzyme with the nature-like ADT-bridged 2Fe_H_-cluster.^27^ As HydA1 is smaller than CpI, a better accessibility of the active site from the protein surface might promote undirected proton transfer. This could enable slow H_2_ production even though the directed proton transfer *via* the amine of the 2Fe_H_-cluster is disrupted.

**Fig. 1:**
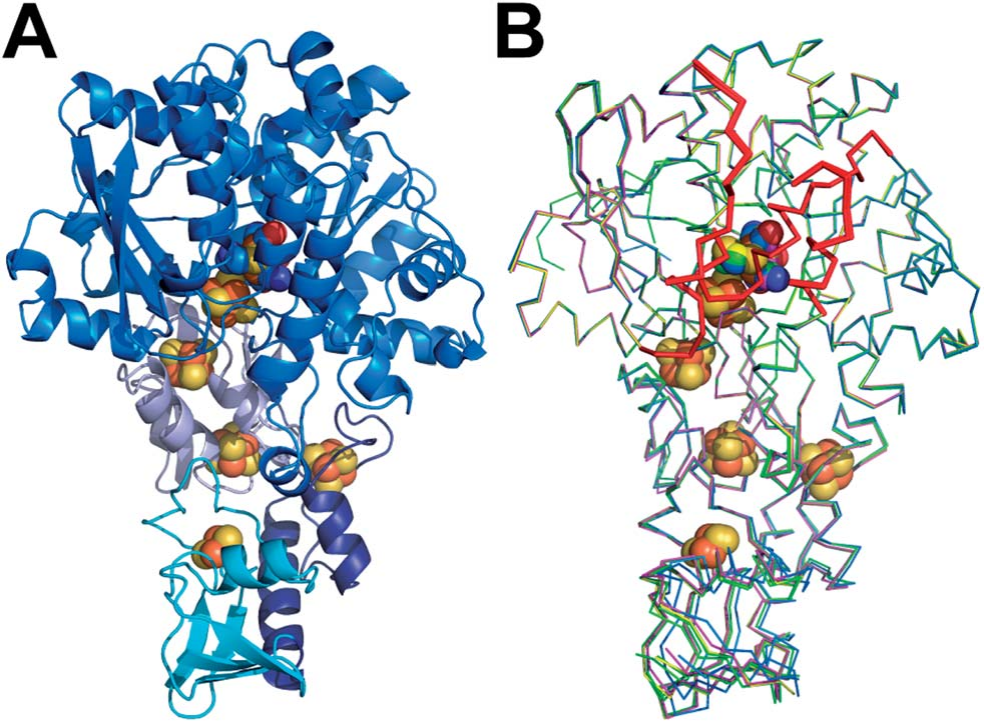
Structures of unmaturated and semisynthetic [FeFe]-hydrogenases. (A) Cartoon model of [FeFe]-hydrogenase CpI^ADT^ with domains in different hues of blue. (B) Overlay of ribbon models of [FeFe]-hydrogenases apoCpI (cyan), CpI^ADT^ (marine), CpI^PDT^ (magenta), CpI^ODT^ (green) and CpI^SDT^ (yellow). H domain regions significantly different to apoHydA1 indicated as thicker ribbon and in red in B. FeS cluster atoms depicted as spheres and colored according to element (Fe = brown, S = beige, O = red, N = blue, C in color of respective protein) in A and B.

All forms of CpI were crystallized under strictly anaerobic conditions and the crystal structures of both CpI^ADT^ and apoCpI were solved with molecular replacement using the known structure of active, native CpI^3^,^30^ as a search model and refined to 1.63 Å and 1.60 Å resolution respectively (Fig. 1, Table 1). CpI^ADT^ was subsequently used as a search model during molecular replacement to determine the structures of CpI^PDT^, CpI^ODT^ and CpI^SDT^ at 1.82 Å, 1.73 Å and 1.93 Å resolution respectively (Fig. 1, Table 1). In contrast to already known structures of native CpI,^3^,^30^,^39^ the space group of the crystals was *P*2_1_ for all five enzymes and the asymmetric units each contained two nearly identical molecules. Of these two molecules, one possesses a more flexible N-terminal domain (residues 1–90), but at the same time a more rigid active site and thus yields a more reliable electron density in the important H-domain in all structures. This becomes evident through the slightly lower temperature factors around the active site when compared to the second molecule. Accordingly figures and values given in the text were taken from the former molecule (chain B) if not stated otherwise, while the complete values for both chains of all structures can be found in the ESI.†

**Table 1:** Crystal data and refinement statistics

	apoCpI	CpI^ADT^	CpI^PDT^	CpI^ODT^	CpI^SDT^
**A. *Crystallographic data***					
X-ray source	SPring8-BL44XU	SLS-PXII	SLS-PXII	SLS-PXII	SLS-PXII
Space group	*P*2_1_	*P*2_1_	*P*2_1_	*P*2_1_	*P*2_1_
Unit-cell parameters					
*a* (Å)	90.06	91.34	87.47	89.66	89.83
*b* (Å)	71.81	73.65	72.07	72.45	73.13
*c* (Å)	103.31	103.88	102.71	102.94	103.04
Wavelength (Å)	0.900	0.978	0.979	0.979	0.979
Resolution range (Å)	50.00–1.60	48.365–1.63	47.58–1.82	47.77–1.73	48.03–1.93
	(1.63–1.60)^*a*^	(1.67–1.63)	(1.87–1.82)	(1.77–1.73)	(1.98–1.93)
Total reflections	645 133 (31 520)	3 729 315 (129 491)	760 236 (56 288)	929 911 (70 575)	674 928 (47 804)
Unique reflections	172 056 (8519)	170 277 (12 470)	112 496 (8270)	136 702 (10 132)	99 824 (7353)
Completeness (%)	99.7% (99.5%)	99.9% (99.7%)	99.9% (100.0%)	100.0% (100.0%)	99.9% (99.9%)
*R*_meas_ (%)	5.2% (56.5%)	8.7% (109.1%)	14.3% (98.8%)	8.7% (83.1%)	15.8% (93.6%)
*I*/*σ*(*I*)	36.8 (3.2)	20.6 (2.2)	9.6 (2.0)	14.1 (2.1)	9.9 (2.1)
Correlation coefficient (CC 1/2)^*b*^	— (—)	99.9 (87.1)	99.7 (77.9)	99.9 (84.1)	99.6 (68.8)

**B. *Refinement statistics***					
PDB code	4XDD	4XDC	5BYR	5BYQ	5BYS
Resolution (Å)	1.60	1.63	1.82	1.73	1.93
*R*_work_	0.14	0.15	0.16	0.15	0.16
*R*_free_	0.17	0.18	0.19	0.18	0.19
No. atoms (except H)	10 548	10 385	9859	9978	9991
Protein	9050	9047	8893	8982	8903
Ligand	72	106	106	106	106
Solvent/ion	1426	1232	860	890	982
RMSD from ideal					
Bond lengths (Å)	0.006	0.013	0.018	0.009	0.014
Bond angles (°)	0.95	1.28	1.48	1.07	1.28
Ramachandran plot					
Most favored (%)	97.67	96.90	96.32	97.23	96.59
Additionally allowed (%)	2.33	3.02	3.68	2.77	3.41
Outliers (%)	0.00	0.09	0.00	0.00	0.00
B factors					
Overall	35.0	39.0	32.0	35.0	32.0
2Fe_H_ cavity (chain A/chain B)	19.5/22.7	24.5/22.0	19.0/16.8	22.3/19.3	18.6/17.3
2Fe_H_ (chain A/chain B)	—/—	23.3/20.7	19.3/17.6	23.1/20.2	18.5/17.3
Average occupancy of 2Fe_H_ (chain A/chain B)	—/—	0.98/0.95	0.94/0.96	0.93/0.96	0.98/0.97

^*a*^Numbers in parenthesis represent values for the highest resolution bin.

^*b*^Correlation coefficient CC (1/2) as defined in Karplus and Diederichs 2012.^55^

As *in vitro* maturation of apo-[FeFe]-hydrogenases with synthetic [2Fe] cofactors was described only recently,^2^,^26^ we considered the exact structure of the 2Fe_H_-cluster and its environment in the semisynthetic enzyme to be of considerable interest. To minimize model bias of electron density in the active site cavity before starting to refine the 2Fe_H_-subcluster, at least two rounds of refinement of each structure were performed without a 2Fe_H_-cluster in the models.

Subsequently, starting models of the 2Fe_H_-subcluster based on the structure of native CpI^30^ with optimized geometry but adapted composition of the dithiolato moiety,^5^ were used. Restraints were applied to all bond distances in the subcluster. We additionally restrained the angles defining the positions of the CO and CN^–^ ligands. The position of the bridging CO was not restrained due to its reported flexibility^5^ depending on the redox state of the enzyme. Final models of the 2Fe_H_-subclusters were verified by inspection of composite omit maps.

### Presumed maturation channel closed in apoCpI crystal structure

**Fig. 2:**
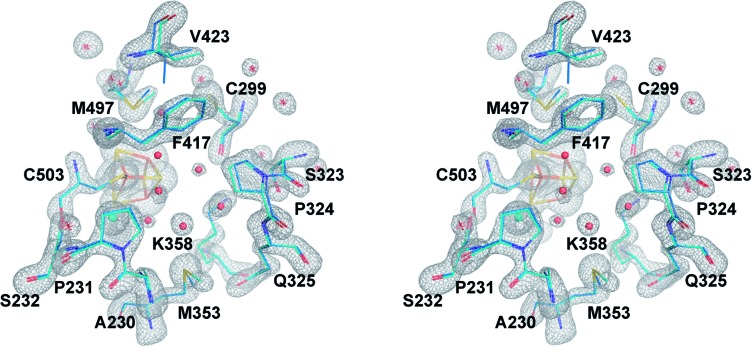
The central cavity of apoCpI and CpI^ADT^. Stereo view of a stick model of the central cavity of apoCpI (carbon atoms in cyan) with *F*_o_ – *F*_c_ simulated annealing omit map contoured at 4σ in the identical orientation as Fig. 3. A stick model of amino acids lining the central cavity of CpI^ADT^ is superposed (carbon atoms in marine). Numbering of amino acids as in the structure of native CpI. Small spheres indicate water atoms (red) and chloride ion (cyan) not present in CpI^ADT^.

The crystal structure of apoCpI reported here is strikingly similar to structures of active CpI, both native and semisynthetic (Fig. 1). The overall RMSD of the backbone atoms of apoCpI and native CpI^30^ is as low as 0.3 Å, while apoCpI and CpI^ADT^ display an RMSD of 0.4 Å over all backbone atoms (Table S1†). Significant differences in side-chain orientation are mainly limited to surface exposed residues with V423 being a notable exception (Fig. 2). This residue in the central cavity is adapting a different rotamer, supposedly stabilized by one of the water molecules in the binding pocket for 2Fe_H_. As demonstrated earlier,^31^ the structure of apoHydA1 from *C. reinhardtii* lacking the 2Fe_H_-subcluster shows overall great similarity to the structure of the H-domain of CpI^3^ and DdH^29^ with regard to the backbone geometry, but exhibits regions of pronounced differences. Amongst these differences is a channel from the surface to the site of the 2Fe_H_-subcluster, which is only present in apoHydA1.^31^ Three regions, which can be understood as plug, lock and lid, block the channel in all known structures of active [FeFe]-hydrogenases (Fig. S1B†), while they are remote in apoHydA1. They presumably shift to close the channel and complete the first sphere of amino acid residues around the H-cluster upon integration of the 2Fe_H_-subcluster in HydA1 (ref. 31) (Fig. S1A†). However, there has neither been a structure of a maturated [FeFe]-hydrogenase of the short chlorophyta type nor of an unmaturated bacterial type enzyme, which would have allowed for a direct comparison.

The structure of apoCpI presented here shows the three regions 405–423 (“plug”), 437–453 (“lid”) and 529–540 (“lock”) clearly in a “closed” conformation nearly identical to active CpI (Fig. 1). Prominently, F417 in direct contact to the 2Fe_H_-subcluster shows minimal deviation in apoCpI when compared to CpI^ADT^ (Fig. 2), while it is moved by 15 Å in apoHydA1. Washed and subsequently dissolved crystals of apoCpI could be maturated with the synthetic ADT-bridged [2Fe] cluster to an activity of 1250 nmol H_2_/min/crystal, reassuring that the reported closed structure of apoCpI is not a dead-end conformation. This suggests an equilibrium between a “closed” and an “open” state in apoCpI, of which only the former readily crystallizes. Within the regions with striking deviation between apoHydA1 and apoCpI, several glycine residues can be identified, which are highly conserved in a recent sequence alignment of all known [FeFe]-hydrogenase sequences^40^ (Table S2†). These amino acids could function as hinges, as for some of them the “open” or “closed” conformation respectively would imply dihedral angle combinations commonly found only for glycine residues^41^ (Table S2†). Their high degree of conservation thus is another hint that an open and closed form of all [FeFe]-hydrogenases exists.

### Rigid cavity in apoCpI forces the [2Fe] complex to move into its active conformation

**Fig. 3:**
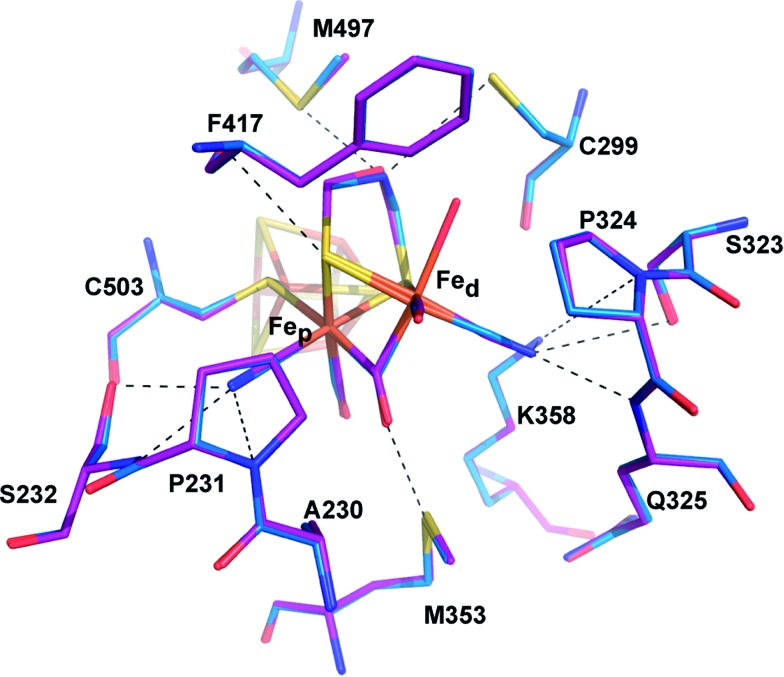
Comparison of the active site of CpI^ADT^ and native CpI. Stick model of the environment of the 2Fe_H_-subcluster of CpI^ADT^ (carbon atoms in marine) superposed to a stick model of native CpI (PDB ID ; 3C8Y)^30^ (carbon atoms in magenta). Dashed lines indicate potential interactions between 2Fe_H_ and the protein listed in Table S4.† Numbering of amino acids as in the structure of native CpI.

Being devoid of the 2Fe_H_-cluster, the active site binding pocket of apoCpI is occupied by seven water molecules and a chloride ion (Fig. 2) instead. Note that this leaves a water filled cavity of roughly 10 Å diameter in the center of the protein. Nonetheless, residues which are assumed to interact with the cofactor in the active enzyme are shifted only very slightly by 0.1–0.3 Å towards a narrower cavity (Table S3,† Fig. 2) and show the same low temperature factors as most of the H-domain (Table 1). This exemplifies how the amino acids of seven distinct protein parts (around amino acids 231, 299, 324, 353, 417, 497 and 503; Fig. 3) coordinate to form a rigid central cavity, perfectly positioned to arrange the ligands of a [2Fe] cluster in its center.

### Crystal structure of semisynthetic CpI^ADT^ reveals native-like structure and open coordination site

Superposition of the structure of semisynthetic CpI^ADT^ and the best available crystal structure of native CpI^30^ results in nearly identical structures with an RMSD of 0.3 Å for the main chain atoms. Even with regard to the side chain atom conformations, significant differences between CpI^ADT^ and the native CpI can only be found in several surface exposed residues, which is surprising given the considerable differences in the crystal packing.

**Fig. 4:**
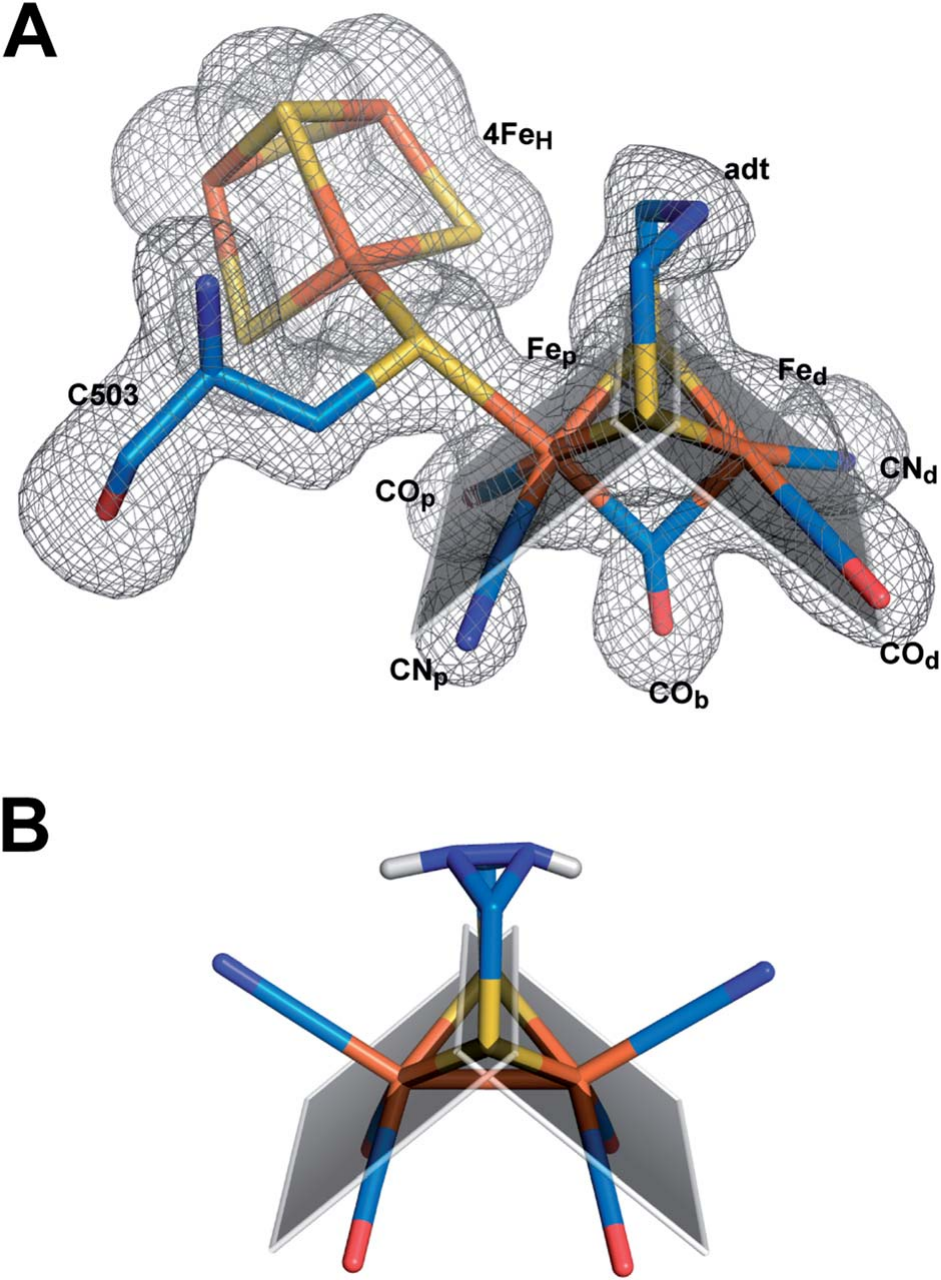
Structure of semisynthetic H-cluster and structural changes in ligand coordination upon integration of 2Fe_H_. (A) Stick model of the H-cluster of CpI^ADT^ colored according to element with *F*_o_ – *F*_c_ simulated annealing omit map contoured at 3.5σ. (B) Stick model of the crystal structure of Fe_2_[μ-(SCH_2_)_2_NH](CN)_2_(CO)_4_^2–^ (ref. 37). The planes in A and B are drawn through the sulfur atoms of the [2Fe] complexes and one of the two Fe atoms each to clarify the coordination geometry of the Fe ligands.

When comparing the important cofactor–peptide interactions in native CpI and the structure of CpI^ADT^, the distances between the atoms of 2Fe_H_ and their respective interaction partners in the protein environment show a maximum deviation of 0.17/0.13 Å and an average deviation of 0.06/0.05 Å for chain A/chain B (Fig. 3, Table S4†). This is well within the experimental error of crystal structure analysis at the given resolution. Moreover the synthetic 2Fe_H_-cluster itself in the structure of CpI^ADT^ compares very well to the *in vivo* synthesized version in native CpI^30^ (Fig. 3) and the [FeFe]-hydrogenases DdH^29^ and HydA1 (data from XANES/EXAFS)^42^ within the error of crystal structures of macromolecules (Table S5†). This finding confirms data from FTIR and EPR spectroscopic studies, which showed excellent agreement of native and semisynthetic protein for the small [FeFe]-hydrogenase HydA1 from *Chlamydomonas reinhardtii*.^26^,^28^ For [FeFe]-hydrogenases with additional N-terminal domains like CpI or DdH, a bridging conformation of one CO ligand is assumed to occur only in the H_ox_ or CO inhibited state.^5^,^21^,^29^ In our structure the CO ligand between the Fe atoms is positioned at an angle of 114°/132° (chain A/chain B) between Fe_d_–C–O (Table S5,† Fig. 4), which does not indicate terminal binding of the CO to Fe_d_ as previously published for a structure of reduced DdH.^5^ Thus we understand the here reported structure of CpI^ADT^ to be mainly in the H_ox_ state. While in earlier structures of CpI^3^,^30^ a region of low but significant electron density next to Fe_d_ was assigned as a water molecule in this particular redox state, in the structure described here the H-cluster of both chains clearly features one coordination site on Fe_d_ devoid of electron density (Fig. 4).

A comparison of the crystal structures of the synthetic ADT-bridged [2Fe] complex^37^ before and after integration into the protein environment as 2Fe_H_ illustrates the distortions that the protein forces upon the [2Fe] complex (Fig. 4). An Fe–S–Fe bridge to the 4Fe_H_-cluster is formed and, as demonstrated earlier, one CO ligand is lost during the process of activation.^26^ Another CO ligand shifts into a bridging position between the two Fe atoms and the CO/CN^–^ ligands move into an octahedral coordination at each Fe with nearly perpendicular equatorial planes (Fig. 4). This conformation has been attributed a crucial role in allowing the mixed Fe(i)–Fe(ii) valency of the H_ox_ state within the catalytic cycle, which is difficult to achieve in isolated [2Fe] clusters.^43^ Additionally the new conformation features the open coordination site at Fe_d_*trans* to the bridging CO (Fig. 4). This promotes regioselectivity of H_2_ binding or hydride formation close to the amine in the ADT-bridge, which is believed to be crucial for the mechanism.^16^,^44^

### 2Fe_H_-subsite structure remains unaltered upon changes in the dithiolato bridge

**Fig. 5:**
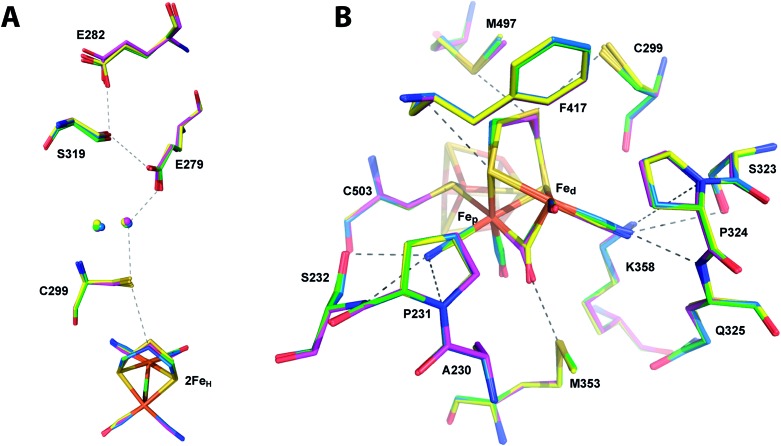
Comparison of the catalytically important amino acids in CpI derivatives. Stick models of the potential proton transfer pathway (A) and the environment of the 2Fe_H_-subcluster (B) of CpI^ADT^ (carbon atoms in marine) superposed to stick models of CpI^PDT^ (magenta), CpI^ODT^ (green) and CpI^SDT^ (yellow). Dashed lines indicate potential proton transfer interactions or potential interactions of 2Fe_H_ with the protein as listed in Table S4.† Numbering of amino acids as in the structure of native CpI.

The structures of all three CpI derivatives with non-natural 2Fe_H_-subsites superpose very well with each other and the native CpI, apoCpI and CpI^ADT^ with RMSD's for Cα atoms between 0.2 Å and 0.5 Å (Table S1†). Comparison of the exact positions of amino acids supposedly involved in enzyme function, *e.g.* amino acids in the proton transfer pathway or around the active site, yielded little differences within the limits of exactness of macromolecular crystallography (Fig. 5). The average RMSDs of all atoms of selected amino acids were as low as 0.08–0.11 Å when comparing the non-natural derivatives with CpI^ADT^. As significant differences in the degree of maturation were observed for semisynthetic HydA1 with non-natural 2Fe_H_ clusters,^27^ we allowed variation of the occupancies of the atoms of the 2Fe_H_-subclusters during refinement. According to this rough estimate more than 90% of the molecules in the crystals contained the 2Fe_H_-subsite (Table 1). Even though the effects of partial occupancy and temperature factor are hardly discernible at the given resolution, we expect these results to be a good lower limit as the calculated temperature factors of the 2Fe_H_-clusters and the surrounding amino acids agree well (Table 1). As apoCpI crystallizes in a nearly identical structure and an isomorphous unit cell we assume that the high occupancy of the 2Fe_H_-clusters does not result from positive selection during crystal formation, but illustrates the effectiveness of *in vitro* maturation of CpI with the chosen [2Fe] clusters.

**Fig. 6:**
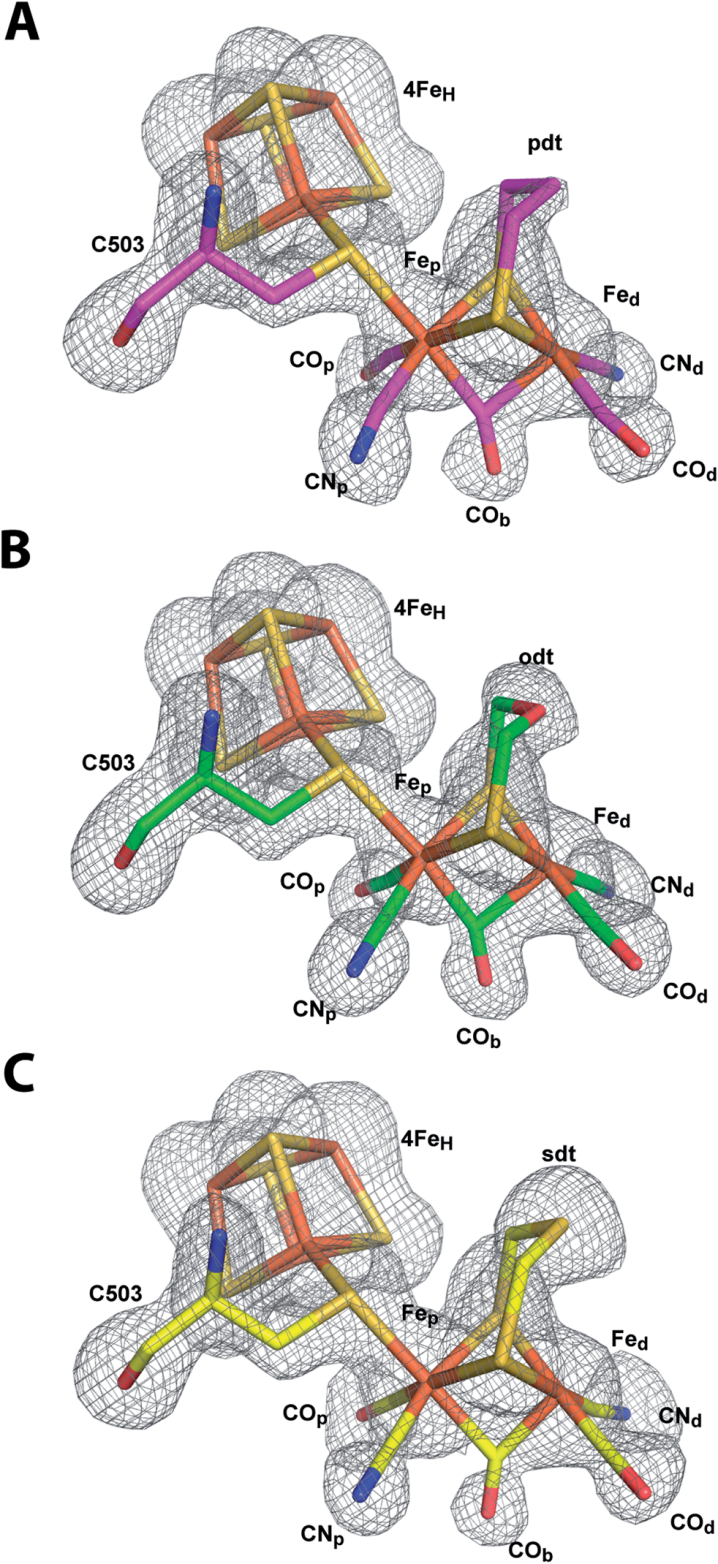
Models of the H-cluster of non-native CpI derivatives. Stick models of (A) CpI^PDT^ (carbon atoms in magenta), (B) CpI^ODT^ (carbon atoms in green) and (C) CpI^SDT^ (carbon atoms in yellow) with *F*_o_ – *F*_c_ simulated annealing omit maps contoured at 3.5σ.

For the ODT-bridged 2Fe_H_-subsite in HydA1 a less pronounced bridging character of CO_b_ compared to other semisynthetic HydA1 enzymes was reported according to FTIR data of the “as isolated” state.^27^ A very similar state represented a minor part of the mixed population of HydA1 with SDT-bridged 2Fe_H_-subcluster in the same study. We found an angle of 145° between Fe_d_–C–O of the CO_b_ ligand in CpI^SDT^ which indicates a more terminal than bridging character, but the other structures including CpI^ODT^ reveal angles suggesting a bridging CO (Fig. 6, Table S5†).

Because of the above discussed effects of redox state changes on the CO_b_ ligand, a potential dependent FTIR based investigation of the CpI enzyme derivatives would be needed to clarify if this is merely an effect of the redox state at the point of crystal mounting or inherent to the different dithiolato bridge. A detailed comparison of distances between the atoms of the 2Fe_H_ subsite and the surrounding amino acids indicates a slightly different position of Fe_d_ within the cavity for CpI^PDT^ 0.1 Å closer to Ala 230 and further away from Cys 299 (Table S4†). Besides this, small differences in the dithiolato bridge can be observed. While the bridgehead atom is leaning about 0.2 Å further away from Met 497 in the inactive CpI derivatives, the sulfur atom of Cys 299 is pushed back to keep roughly the van-der-Waals distance to the bridgehead atom of the dithiolato bridge in the three structures (Fig. 5B, Table S3†). However, the position and geometry of the non-natural 2Fe_H_-clusters do not show any large differences (Fig. 5 and 6, Table S5†) when compared to native CpI or CpI^ADT^ and thus do not offer a clear structural explanation for the impaired activity.

For CpI^PDT^ this is in line with a recent ENDOR and HYSCORE study of HydA1 containing a PDT-bridged 2Fe_H_-subcluster, which showed very similar spectra in comparison to *in vivo* maturated DdH.^45^ DFT calculations performed for ADT-bridged, PDT-bridged and ODT-bridged 2Fe_H_-subclusters in CpI also resulted in very similar geometries^30^ (Table S5†). Remarkably, there is no significant electron density in our structures close to Fe_d_ at the postulated site of H_2_ binding in any of the 2Fe_H_-subclusters (Fig. 6). Thus binding of an inhibitor to this open coordination site can be ruled out as cause for the quantitative loss of activity. For HydA1 with the PDT-bridged 2Fe_H_ cluster no binding of CO to the active site was observed in a recent FTIR based study.^28^ Our structure of CpI^PDT^ rules out a rearrangement in the neighboring amino acids as explanation for this behavior. However, once an inhibitory CO is bound to Fe_d_, the distance between the central atom of the dithiolate bridge and the oxygen of CO was reported to be as close as ∼2.5 Å.^5^ While the single hydrogen of an amine bridgehead proposedly points towards C299 and thus away from Fe_d_, the PDT's central methyl group might considerably obstruct binding of CO to Fe_d_ through its hydrogen atoms not visible in X-ray crystallography at the given resolution.

## Conclusions

We herein report the structure of the [FeFe]-hydrogenase CpI from *Clostridium pasteurianum* in the unmaturated apo-form and the first high resolution structure of a fully active semisynthetic [FeFe]-hydrogenase along with the structures of three non-natural inactive derivatives of this [FeFe]-hydrogenase with changes in the inorganic active site. Surprisingly, the unmaturated apoCpI crystallizes in an overall conformation like the maturated enzyme and not similar to the structure of unmaturated HydA1. The high degree of rigidity of the amino acids in proximity to the H-cluster even in the absence of the 2Fe_H_-subcluster demonstrates how the protein structure is designed to force the 2Fe-cofactor into its highly active form. Semisynthetic CpI^ADT^ shows a nearly identical conformation when compared to the native enzyme including the rotated conformation of the 2Fe_H_ cofactor with octahedral geometry at both Fe atoms. Unlike previous structures of CpI the structure of CpI^ADT^ presented here displays a completely unoccupied open coordination site at Fe_d_. Non-natural derivatives of the 2Fe_H_ subsite with changes in the central atom of the dithiolato bridge can well be incorporated into apoCpI as already reported for apoHydA1, but do not lead to H_2_ evolution activity. The structures of the protein matrix of CpI^PDT^, CpI^ODT^ and CpI^SDT^ show no clear differences to the highly active CpI^ADT^. Despite their divergence in activity all four different 2Fe_H_-subclusters investigated in this study are adapted in their conformation to the protein matrix when compared to the structures of the free [2Fe] complexes and take up the same typical structure. The proposed site of H_2_ oxidation at Fe_d_ is unoccupied in all structures reported here, which excludes inhibitor binding or steric hindrance as reasons for impaired activity. The structural information gained in this study in combination with previously reported FTIR^27^,^28^ and EPR^45^ spectroscopic data of inactive active site variants of [FeFe]-hydrogenases underline the central role the chemistry of the dithiolato bridge plays for enzyme activity. Once the protein environment has forced the iron ligands into the typical conformation, which is the basis of H_2_ evolution, it is solely the reactivity of the central amine, which induces enzyme activity.

## Experimental section

ApoCpI was expressed with a C-terminally fused strep-tagII in E.c. BL21(DE3) ΔiscR^46^ under anaerobic conditions as described earlier^47^ without coexpression of [FeFe]-hydrogenase specific maturases. Protein purification was achieved by strep-tactin affinity chromatography under strictly anaerobic conditions^48^ with a 10 mM Tris–HCl buffer with pH 8.0 and 2 mM NaDT and purity was assessed by SDS-PAGE.

[Fe_2_[μ-(SCH_2_)_2_NH](CN)_2_(CO)_4_][Et_4_N]_2_ was synthesized as reported earlier.^2^ As the purification of [Fe_2_[μ-(SCH_2_)_2_NH](CN)_2_(CO)_4_][Et_4_N]_2_ by washing with hexane^34^ did not result in clean product, the recently described purification procedure for [Fe_2_[μ-(SCH_2_CH_2_CH_2_S)](CN)_2_(CO)_4_][Et_4_N]_2_ (ref. 2) was adopted for [Fe_2_[μ-(SCH_2_)_2_NH](CN)_2_(CO)_4_][Et_4_N]_2_.

[Fe_2_[μ-(SCH_2_CH_2_CH_2_S](CN)_2_(CO)_4_][Et_4_N]_2_, [Fe_2_[μ-(SCH_2_)_2_S](CN)_2_(CO)_4_][Et_4_N]_2_ and [Fe_2_[μ-(SCH_2_)_2_O](CN)_2_(CO)_4_][Et_4_N]_2_ were synthesized according to literature procedures^33^,^35^,^36^,^38^ and purity of each sample was checked by ^1^H NMR and IR spectroscopy. Samples were stored at –35 °C under inert atmosphere to avoid any decomposition of the artificial cofactors.

Maturation of apoCpI to CpI^ADT^, CpI^PDT^, CpI^ODT^ or CpI^SDT^ with a 10 fold excess of [Fe_2_[μ-(SCH_2_)_2_X](CN)_2_(CO)_4_][Et_4_N]_2_ was achieved in a 0.1 M K_2_HPO_4_/KH_2_PO_4_ buffer system at pH 6.8 with 2 mM NaDT as described earlier^26^ at RT for 1 hour to ensure complete maturation of the sample. The semisynthetic enzymes were subsequently cleaned from leftover [2Fe] complex and buffered again into a 10 mM Tris–HCl buffer with pH 8.0 and 2 mM NaDT by use of a NAP™ 5 (GE Healthcare) size exclusion chromatography column. Enzyme preparations were concentrated using Amicon Ultra centrifugal filters 30 K (Millipore) under anaerobic conditions. Success of maturation and quality of purified protein samples of CpI^ADT^ were determined by testing their H_2_ production activity *in vitro* with methylviologen as electron donor using an established method.^49^ To test for catalytic activity of the non-native semisynthetic enzymes, the same method was applied and additional measurements with 10 fold increased protein amount were conducted to lower the limit of detection.

Box-like protein crystals of apoCpI and the semisynthetic hydrogenases were obtained with PEG 3000 or PEG 4000 as precipitant using the hanging drop or sitting drop vapor diffusion method at 277 K under anaerobic conditions within 2–4 days when mixing reservoir solution 1 : 1 with protein solution (10 mg ml^–1^). The crystallization conditions for the selected crystals of apoCpI were 12% PEG 3000, 0.1 M MES pH 6.5, 0.2 M MgCl_2_ in a sitting drop vapor diffusion experiment and cryo-protection was achieved with a final concentration of 15% glycerol in 15% PEG 3000, pH 6.5, 0.2 M MgCl_2_. CpI^ADT^ crystals selected for diffraction experiments were grown in 11% PEG 4000, 0.1 M MES pH 7.0, 0.2 M MgCl_2_ in a hanging drop experiment and protected against formation of ice crystals with paraffin oil. Crystals of the non-native semisynthetic enzymes were grown by hanging drop vapor diffusion using 0.1 M MES pH 6.0, 0.4 M MgCl_2_ and a total of 40% v/v of PEG4000 and glycerol to avoid the need of additional cryo-protection during crystal mounting. In detail the reservoir solutions contained 15% PEG 4000, 25% glycerol for CpI^PDT^, 19% PEG 4000, 21% glycerol for CpI^ODT^ and 21% PEG 4000, 19% glycerol for CpI^SDT^.

Maturation capability of crystallized apoCpI was tested by washing a crystal in three fresh drops of its reservoir solution followed by dissolution of the crystal in cold 0.1 M K_2_HPO_4_/KH_2_PO_4_ buffer at pH 6.8 with 2 mM NaDT under strictly anaerobic conditions. Maturation was started by addition of 1.5 pmol Fe_2_[μ-(SCH_2_)_2_NH](CN)_2_(CO)_4_[Et_4_N]_2_ in 0.1 M K_2_HPO_4_/KH_2_PO_4_ buffer, pH 6.8, and allowed to proceed for 1 h at 4 °C. Subsequently the mixture was transferred completely into a solution of methylviologen, NaDT and phosphate buffer as for standard tests for H_2_ evolution activity and treated accordingly.^49^

Mounting of protein crystals into CryoLoops™ (Hampton Research) and subsequent flash-freezing in liquid N_2_ was performed under strictly anaerobic conditions at 298 K. Diffraction data were collected at 100 K at beamline BL44-XU at SPring-8 (Hyogo, Japan) and beamline PXII at the SLS (Villigen, Switzerland) and the data were processed using the software package HKL2000 (ref. 50) and XDS^51^ for apoCpI and the semisynthetic hydrogenases, respectively. Molecular replacement and structure optimization were performed with the software packages CCP4 (ref. 52) (apoCpI and CpI^ADT^) and PHENIX^53^ (CpI^PDT^, CpI^ODT^ and CpI^SDT^) and Coot.^54^ At least two final refinement runs were conducted with PHENIX on all structures to improve comparability of the final models. In order to estimate the occupancy of the 2Fe_H_-cluster in the structures of CpI^ADT^ and other derivatives, we applied a partial occupancy refinement at the final stage of PHENIX refinement. Simulated annealing omit maps were calculated with PHENIX, omitting the H-cluster with the bridging cysteine residue and the residues around the central cavity as well as all atoms within the central cavity for the semisynthetic [FeFe]-hydrogenases and apoCpI, respectively.

## Funding sources

This work was supported in part by an International Joint Research Promotion Program, Osaka University, (G.K, T.H.); the Cabinet Office of Japan, through the Funding Program for Next Generation World-Leading Researchers (NEXT Program) (GS016, to G.K.) and the Studienstiftung des deutschen Volkes (J.E.). U.-P.A was supported by the Fonds of the Chemical Industry (Liebig grant) and the Deutsche Forschungsgemeinschaft (Emmy Noether grant AP242/2-1). T.H. thanks the Volkswagen Foundation (LigH2t). The authors are also grateful to the Cluster of Excellence RESOLV (EXC1069) funded by the Deutsche Forschungsgemeinschaft (DFG) for financial support.

## Conflict of interest

The authors declare no competing financial interest.

## Accession numbers

The coordinates and structure factors for all structures were deposited with the Protein Data Bank under the following accession numbers: apoCpI: 4XDD, CpI^ADT^: ; 4XDC, CpI^PDT^: ; 5BYR, CpI^ODT^: ; 5BYQ, CpI^SDT^: ; 5BYS.

## Abbreviations

2Fe_H_: [2Fe] subcluster of the H-cluster of [FeFe]-hydrogenases
4Fe_H_: [4Fe4S] subcluster of the H-cluster
ADT: Aza-dithiolate
apoCpI: CpI lacking only the 2Fe_H_-cluster
CpI: [FeFe]-hydrogenase I from *Clostridium pasteurianum*
CpI^ADT^: apoCpI maturated *in vitro* with (Fe_2_[μ-(SCH_2_)_2_NH](CN)_2_(CO)_4_^2–^)
CpI^ODT^: apoCpI maturated *in vitro* with (Fe_2_[μ-(SCH_2_)_2_O](CN)_2_(CO)_4_^2–^)
CpI^PDT^: apoCpI maturated *in vitro* with (Fe_2_[μ-(SCH_2_)_2_CH_2_](CN)_2_(CO)_4_^2–^)
CpI^SDT^: apoCpI maturated *in vitro* with (Fe_2_[μ-(SCH_2_)_2_S](CN)_2_(CO)_4_^2–^)
DdH: [FeFe]-hydrogenase from *Desulfovibrio desulfuricans*
HydA1: [FeFe]-hydrogenase I from *Chlamydomonas reinhardtii*
ODT: Oxo-dithiolate
PDT: Propane-dithiolate
SDT: Sulfur-dithiolate

## Supplementary Material

Supplementary information
