# Supplementary material for: A structural view of synthetic cofactor integration into [FeFe]-hydrogenases
Source: Chem Sci. 2015 Oct 26;7(2):959–68. doi: 10.1039/c5sc03397g (PMC5954619; doi:10.1039/c5sc03397g)
Supplement: Supplementary file 1 [file SC-007-C5SC03397G-s001.pdf]

Electronic Supplementary Material (ESI) for Chemical Science.
This journal is © The Royal Society of Chemistry 2015

A structural view of synthetic cofactor integration into [FeFe]-hydrogenases

Julian Esselborn[a], Norifumi Muraki[b], Kathrin Klein[c], Vera Engelbrecht[a], Nils Metzler-Nolte[c], Ulf-Peter Apfel[c*], Eckhard Hofmann[d], Genji Kurisu[b*], Thomas Happe[a*]

Table S1. RMSD of Cα carbons of the reported structures in Å when superposed to the equivalent chain of CpI^{ADT} or native CpI (3C8Y).

	CpI^{ADT}	native CpI
apoCpI A	0.46	0.23
apoCpI B	0.4	0.32
CpI^{ADT} A	-	0.31
CpI^{ADT} B	-	0.29
CpI^{PDT} A	0.53	0.45
CpI^{PDT} B	0.42	0.36
CpI^{ODT} A	0.44	0.39
CpI^{ODT} B	0.39	0.3
CpI^{SDT} A	0.4	0.38
CpI^{SDT} B	0.42	0.33
native CpI	0.32	0.36

Table S2. Conserved glycine residues, their relative position and their dihedral angles in apoHydA1 and apoCpI.

			apoHydA1		apoCpI	
Residue	RMSD [Å]	Degree of conservation[a]	ϕ (°)	ψ (°)	ϕ (°)	ψ (°)
G245/G412	15.5	90.80%	**86**[b]	**-179**	-63	-27
G251/G418	14.8	87%	**117**	**-174**	-65	-12
G254/G421	7.2	99%	91	2	96	-6
G255/G422	3.7	100%	**99**	**163**	-72	-50
G283/G450	16.0	83%	83	9	**92**	**-176**
G286/G453	1.4	78%	69	15	-70	-35

[a] According to a recent MSA from Winkler et al., 2013.
[b] Values in bold-face indicate angle-combinations only favorable for glycine according to Lovell et al., 2003.

Tabel S3. Position of 2FeH subcluster within the active site cavity in different CpI structures given as distances in Å and angles in °.

Atom 1	Atom 2	native CpI (3C8Y)	apoCpI		CpI^{ADT}		CpI^{PDT}		CpI^{ODT}		CpI^{SDT}	
			chain a	chain b	chain a	chain b	chain a	chain b	chain a	chain b	chain a	chain b
230 CA	299 CA	12.1	12.1	12.1	12.1	12.1	12.1	12.1	12.1	12.1	12.2	12.2
230 CA	2FeH Fed	6.2			6.2	6.1	6.0	6.0	6.1	6.1	6.2	6.1
299 CA	2FeH Fed	6.1			6.2	6.2	6.3	6.3	6.2	6.2	6.2	6.3
353 CA	497 CA	14.6	14.7	14.7	14.6	14.6	14.6	14.6	14.6	14.6	14.6	14.6
353 CA	2FeH Fep	7.0			7.0	7.0	6.9	6.9	7.0	6.9	6.9	7.0
497 CA	2FeH Fep	8.9			8.8	8.9	8.9	8.9	8.9	8.9	8.9	8.9
417 CA	358 CA	14.8	14.5	14.5	14.8	14.8	14.8	14.7	14.7	14.7	14.8	14.8
417 CA	2FeH Fed	6.1			6.1	6.1	6.2	6.1	6.0	6.0	6.1	6.1
358 CA	2FeH Fed	9.0			9.0	9.0	8.9	8.9	8.9	8.9	9.0	9.0
417 CA	2FeH Fep	6.5			6.5	6.5	6.5	6.5	6.4	6.4	6.5	6.5
358 CA	2FeH Fep	8.5			8.5	8.5	8.5	8.5	8.5	8.5	8.5	8.5
230 CB	2FeH O5 (COb)	3.0			3.3	3.1	3.1	3.0	3.1	3.1	3.0	3.0
353 SD	2FeH O5 (COb)	3.2			3.3	3.2	3.2	3.3	3.3	3.3	3.2	3.2
Dihedral C(COb) - Fed - Fep - O (COb)		-3			-1	0	-1	-1	-1	0	0	-1
Dihedral C(COb) - S1 - S2 - O (COb)		-2			-7	-2	-6	-6	-3	-6	3	2

Table S4. Distances between parts of the 2FeH cluster and potential interaction partners in the protein in Å.

			CpI native (3C8Y)	CpI^{ADT}		CpI^{PDT}		CpI^{ODT}		CpI^{SDT}	
				chain a	chain b	chain a	chain b	chain a	chain b	chain a	chain b
adt	C299	SG	3.5	3.5	3.5	3.5	3.6	3.4	3.4	3.5	3.5
	C299	CA	5.0	4.8	4.9	4.7	4.8	4.8	4.8	4.7	4.8
	M497	SD	3.6	3.7	3.7	3.9	3.9	3.9	3.9	4.0	3.9
CNd	S323	OG	3.8	3.9	3.8	3.9	3.8	3.9	3.8	3.9	3.9
	P324	N	3.5	3.5	3.5	3.6	3.4	3.6	3.5	3.5	3.4
	Q325	N	2.9	2.9	2.9	3.0	2.9	2.9	2.9	2.9	2.9
	K358	NZ	2.9	2.8	2.8	2.8	2.8	2.8	2.9	2.9	3.0
CNp	P231	N	3.6	3.6	3.6	3.6	3.6	3.6	3.6	3.6	3.6
	S232	N	3.1	3.1	3.1	3.0	3.1	3.1	3.1	3.1	3.1
		OG	2.8	2.8	2.8	2.9	2.9	2.9	2.9	2.8	2.8
COb	M353	SD	3.2	3.3	3.2	3.2	3.3	3.3	3.3	3.2	3.2
COp	M353	SD	3.5	3.5	3.5	3.6	3.5	3.6	3.5	3.8	3.7
S1	F417	O	3.4	3.5	3.5	3.5	3.6	3.5	3.5	3.5	3.6

Table S5: Distances, angles and dihedral angles of [2Fe2S]-clusters in comparison.

				Cpl^ADT		Cpl^PDT		Cpl^ODT		Cpl^SDT		native Cpl (3C8Y)	DdH (1HFE)	EXAFS of $2Fe_H$ of HydA1[a]	Calculated[b] Cpl^ADT	Calculated[b] Cpl^PDT	Calculated[b] Cpl^ODT	[2Fe2S]-adt complex alone[c]	[2Fe2S]-odt-monocyano complex alone[d]	[2Fe2S]-sdt-hexacarbonyl complex alone[e]	[2Fe2S]-pdt-dicyano complex alone[f]
**Distances [Å]**				chain a	chain b	chain a	chain b	chain a	chain b	chain a	chain b										
Fe_p	S1			2.3	2.3	2.3	2.3	2.3	2.3	2.4	2.4	2.3	2.3	2.22	2.42	2.41	2.43	2.28	2.24		2.28
Fe_p	S2			2.3	2.3	2.3	2.4	2.3	2.3	2.3	2.3	2.3	2.3	2.22	2.41	2.38	2.39	2.29	2.25		2.28
Fe_d	S1			2.3	2.3	2.4	2.3	2.3	2.3	2.4	2.3	2.3	2.3	2.22	2.34	2.33	2.37	2.28	2.27	2.26	2.27
Fe_d	S2			2.4	2.3	2.2	2.3	2.3	2.3	2.3	2.3	2.3	2.3	2.22	2.31	2.33	2.38	2.29	2.26	2.26	2.27
Fe_p	Fe_d			2.6	2.6	2.5	2.5	2.6	2.5	2.6	2.6	2.6	2.6	2.56	2.53	2.53	2.53	2.51	2.51		2.52
S1	C_adt1			1.9	1.9	1.9	1.9	1.9	1.9	1.9	1.9	1.9	1.8		1.88	1.90	1.91	1.87	1.83	1.83	1.83
S2	C_adt2			1.9	1.9	1.9	1.9	1.9	1.9	1.9	1.9	1.9	1.8		1.88	1.91	1.91	1.86	1.84	1.83	1.82
C_adt1	N_adt			1.5	1.5	1.5	1.5	1.5	1.5	1.5	1.5	1.5	1.5		1.51	1.51	1.48	1.43	1.42	1.73	1.54
C_adt2	N_adt			1.5	1.5	1.5	1.5	1.5	1.5	1.5	1.6	1.5	1.5		1.51	1.52	1.47	1.39	1.40	1.75	1.51
Fe_p	C_COp			1.7	1.7	1.7	1.7	1.7	1.7	1.7	1.7	1.7	1.7	1.84	1.67	1.67	1.67	1.76	1.76		1.73
Fe_p	C_CNp			1.8	1.8	1.8	1.8	1.8	1.9	1.8	1.8	1.8	1.8	1.84	1.76	1.76	1.76	1.94	1.92		1.93
Fe_d	C_COb			1.9	1.9	1.9	1.9	1.9	2.0	1.9	1.9	2.0		1.84	2.00	2.00	2.00	1.74			
Fe_d	C_CNd			1.9	1.9	1.9	1.9	1.9	1.9	1.9	1.9	1.9	1.9	1.84	1.99	1.99	1.99	1.94			1.93
Fe_d	C_COd			1.8	1.7	1.8	1.8	1.8	1.8	1.8	1.8	1.8	1.8	1.84	1.82	1.82	1.82	1.75	1.77	1.80	1.74
Fe_p	C_COb			1.8	1.9	1.9	1.9	1.9	1.8	2.1	2.1	1.9		1.84	2.00	2.00	2.00	3.36			
C_COp	O_COp			1.2	1.3	1.2	1.2	1.2	1.2	1.2	1.2	1.2	1.1		1.21	1.21	1.21	1.16	1.15		1.18
C_CNp	N_CNp			1.2	1.2	1.2	1.2	1.2	1.2	1.2	1.2	1.2	1.1		1.20	1.20	1.20	1.15	1.15		1.16
C_COb	O_COb			1.2	1.2	1.2	1.2	1.2	1.2	1.2	1.2	1.2			1.19	1.19	1.19	1.15			
C_CNd	N_CNd			1.1	1.1	1.1	1.1	1.1	1.1	1.1	1.1	1.1	1.1		1.13	1.13	1.13	1.15			1.15
C_COd	O_COd			1.2	1.2	1.2	1.1	1.2	1.2	1.2	1.2	1.2	1.1		1.17	1.17	1.17	1.15	1.14	1.14	1.17
Fe_d	N_adt			3.4	3.4	3.4	3.4	3.3	3.3	3.4	3.4	3.4			3.52	3.44	3.42	3.54	3.27	3.62	3.46
C_adt1	C_adt2			2.5	2.6	2.5	2.5	2.5	2.6	2.3	2.4	2.5			2.60	2.58	2.48	2.44	2.34	2.70	2.52
S1	S2			3.2	3.1	3.2	3.2	3.1	3.1	3.2	3.2	3.1			3.31	3.32	3.35	3.10	3.04	3.09	3.09
**Angles**																					
S1	Fe_p	S2		86	85	88	86	85	84	88	87	87	82		86.4	87.5	87.9	85.5			
S1	C_adt1	N_adt		110	111	124	119	106	107	127	125	117	99		112.5	112.1	112.8	117.2			
Fe_p	C_COp	O_COp		169	171	170	171	170	171	168	169	170	177		166.3	166.3	166.3	177.5			
Fe_p	C_CNp	N_CNp		177	175	169	170	179	178	178	174	179	179		169.8	169.8	169.8	179.4			
Fe_d	C_CNd	N_CNd		172	173	171	170	172	173	171	166	172	177		169.0	169.0	169.0	179.5			
Fe_d	C_COd	O_COd		170	176	174	171	173	175	174	174	175	180		167.3	167.3	167.3	177.9			
S1	Fe_p	C_COp		172	174	171	173	171	169	171	170	174	176		170.6	171.0	172.0	158.3			
S1	Fe_p	C_CNp		86	88	86	87	86	89	84	87	87	81		83.3	84.1	84.6	100.8			
S1	Fe_d	C_COb		87	91	88	88	90	89	94	91	88			92.5	93.0	93.7	158.6			
S1	Fe_d	C_CNd		173	172	173	170	172	172	172	171	174	173		179.8	178.9	178.0	100.7			
S1	Fe_d	C_COd		93	89	89	86	89	92	91	91	91	92		88.5	89.2	89.9	88.0			
Fe_p	C_COb	O_COb		155	144	159	155	147	155	129	132	145			140.0	140.0	140.0				
Fe_d	C_COb	O_COb		114	132	119	121	128	121	151	145	132			140.9	140.9	140.9				
**Dihedrals**																					
Fe_p	Fe_d	S1	C_adt1	-103	-104	-108	-105	-107	-111	-110	-110	-107	-103		-94.8	-97.9	-100.2	-106.1			
Fe_p	Fe_d	S2	C_adt2	103	105	107	101	99	106	103	104	105	105		94.0	97.0	97.1	102.8			
Fe_p	S1	S2	C_COp	-5	-4	-5	-3	-5	-6	-6	-4	-4	-2		-5.3	-5.1	-4.5	-12.7			
Fe_p	S1	S2	C_CNp	-5	-6	-3	-5	-6	-6	-5	-7	-4	-6		-5.1	-5.6	-4.9	41.3			
Fe_d	S1	S2	C_COb	51	48	50	50	47	50	45	47	51			48.1	48.7	47.7	12.4			
Fe_d	S1	S2	C_CNd	-2	-3	-2	-1	-2	-3	0	1	2	3		-0.1	-0.4	-0.8	-40.2			
Fe_d	S1	S2	C_COd	-2	-3	-3	-3	-3	-3	-1	-1	1	-3		-2.6	-1.5	-1.4	13.1			

[a] Lambertz et al., 2014. [b] Pandey et al. 2008. [c] Li and auchfuss 2002. [d] Song et al. 2004 [e] Song et al. 2007 [f] Schmidt et al. 1999

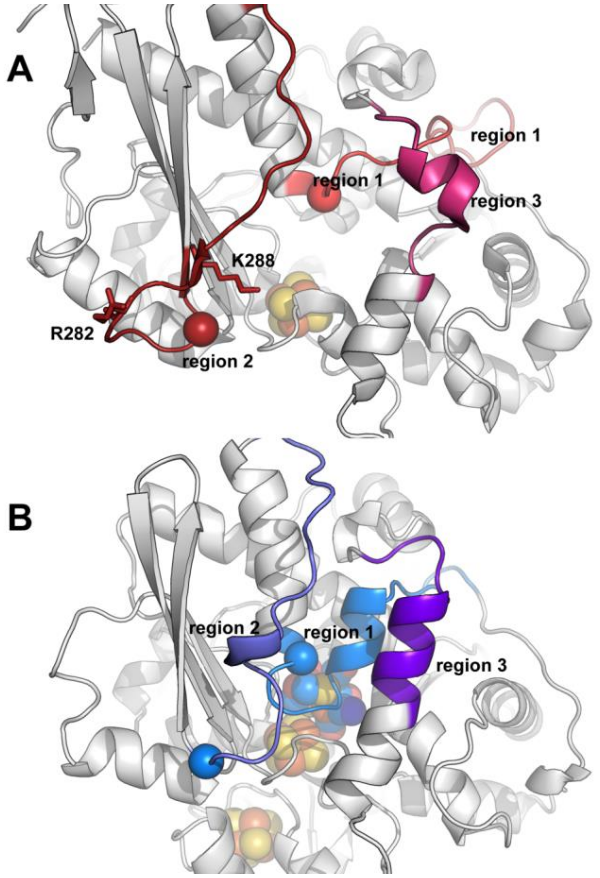

Figure S1: Presumed maturation channel in apoHydA1 and CpI^{MIM}. (A) View along the presumed maturation channel of a cartoon representation of apoHydA1 (PDB ID 3LX4). Regions with high RMSD in comparison to CpI are marked in red tones and labeled as mentioned in the text (region 1: 238-256; region 2:270-288; region 3: 402-413; numbering of 3LX4). Conserved cationic amino acids R282/449 and K288/455 are shown in stick representation (numbering as in 3LX4/3C8Y). (B) Cartoon representation of CpI^{MIM} in the same orientation as apoHydA1 in A with regions of difference marked with blue hues (region 1: 405-423; region 2:437-453 ; region 3: 529-540; numbering as in 3C8Y). Potential glycine hinges G255/422 and G286/453 and FeS clusters are shown as spheres in A and B.

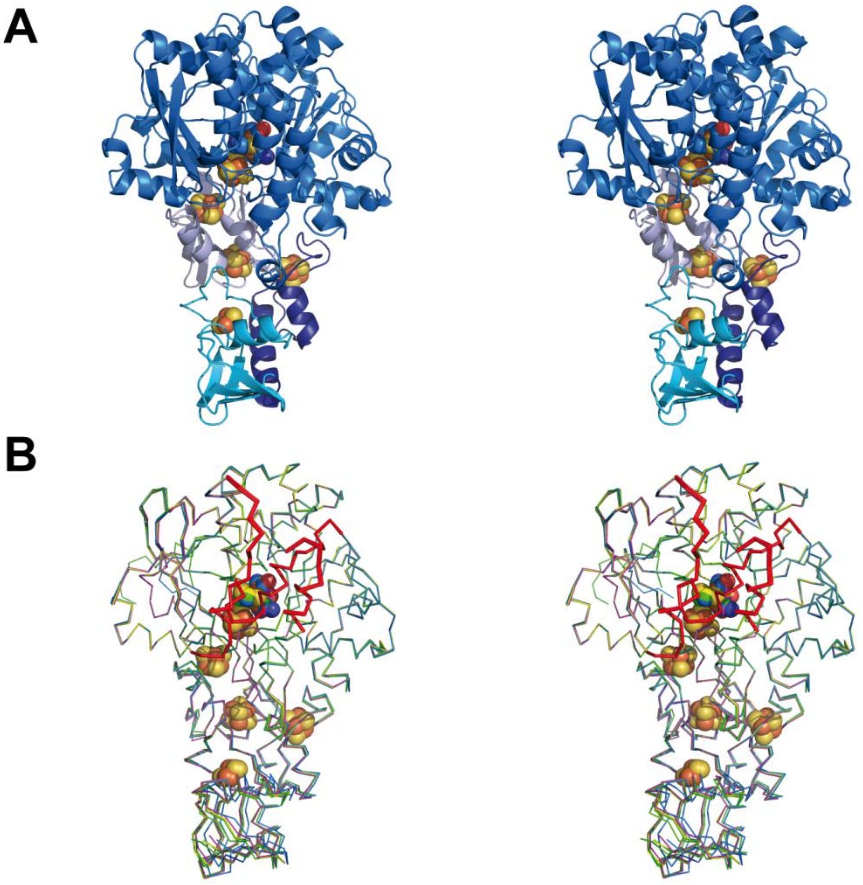

Figure S2: Stereo view of Figure 1: Structures of unmaturated and semisynthetic [FeFe]-hydrogenases.
(A) Cartoon model of [FeFe]-hydrogenase CpI^{ADT} with domains in different hues of blue. (B) Overlay of ribbon models of [FeFe]-hydrogenases apoCpI (cyan), CpI^{ADT} (marine), CpI^{PDT} (magenta), CpI^{ODT} (green) and CpI^{SDT} (yellow). H-domain regions significantly different to apoHydA1 indicated as thicker ribbon and in red in B. FeS cluster atoms depicted as spheres and colored according to element (Fe=brown, S=beige, O=red, N=blue, C in color of respective protein) in A and B.

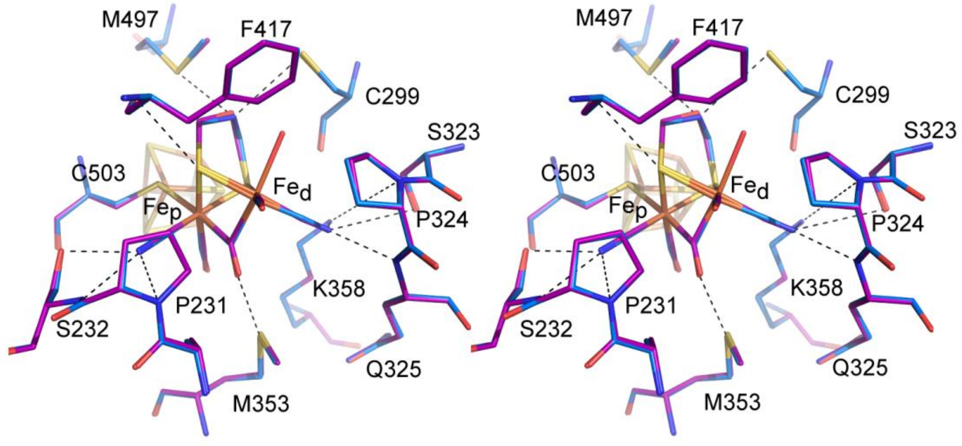

Figure S3: Stereo view of Figure 3: Comparison of the active site of CpI^{ADT} and native CpI. Stick model of the environment of the $2Fe_H$-subcluster of CpI^{ADT} (carbon atoms in marine) superposed to a stick model of native CpI (PDB ID 3C8Y) (carbon atoms in magenta). Dashed lines indicate potential interactions between $2Fe_H$ and the protein listed in table S4. Numbering of amino acids as in the structure of native CpI.

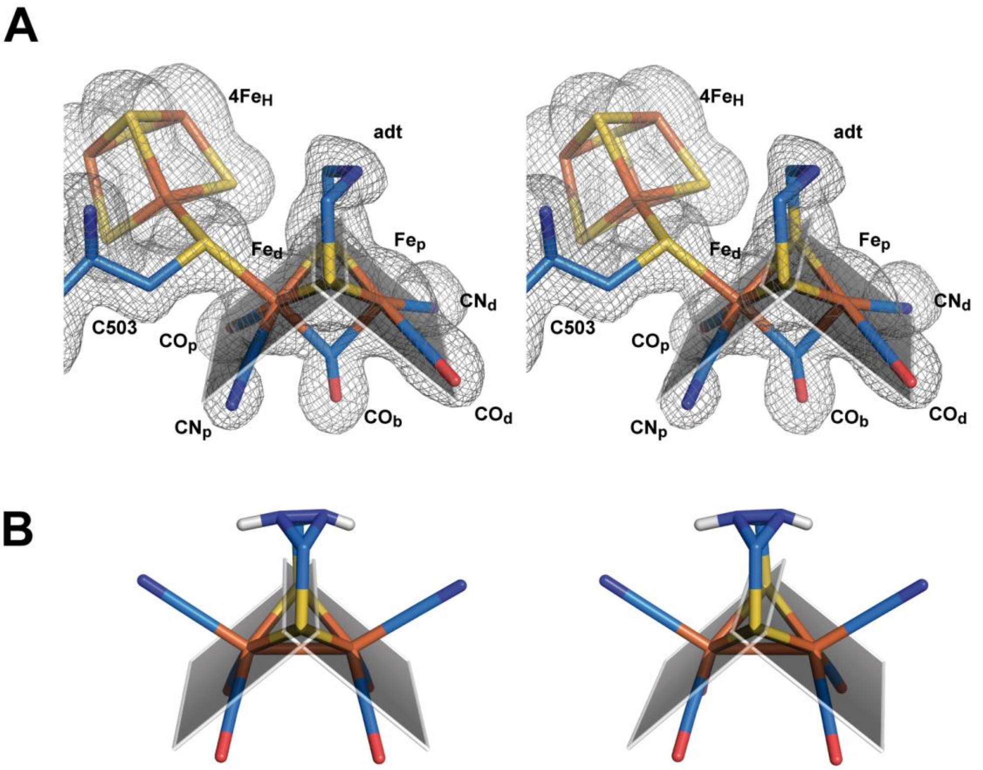

Figure S4: Stereo view of Figure 4: Structure of semisynthetic H-cluster and structural changes in ligand coordination upon integration of $2Fe_H$. (A) Stick model of the H-cluster of CpI^{ADT} colored according to element with F_o-F_c simulated annealing omit map contoured at 3.5σ. (B) Stick model of the crystal structure of $Fe_2[\mu\text{-}(SCH_2)_2NH](CN)_2(CO)_4^{2-}$. The planes in A and B are drawn through the sulfur atoms of the [2Fe] complexes and one of the two Fe atoms each to clarify the coordination geometry of the Fe ligands.

A

B

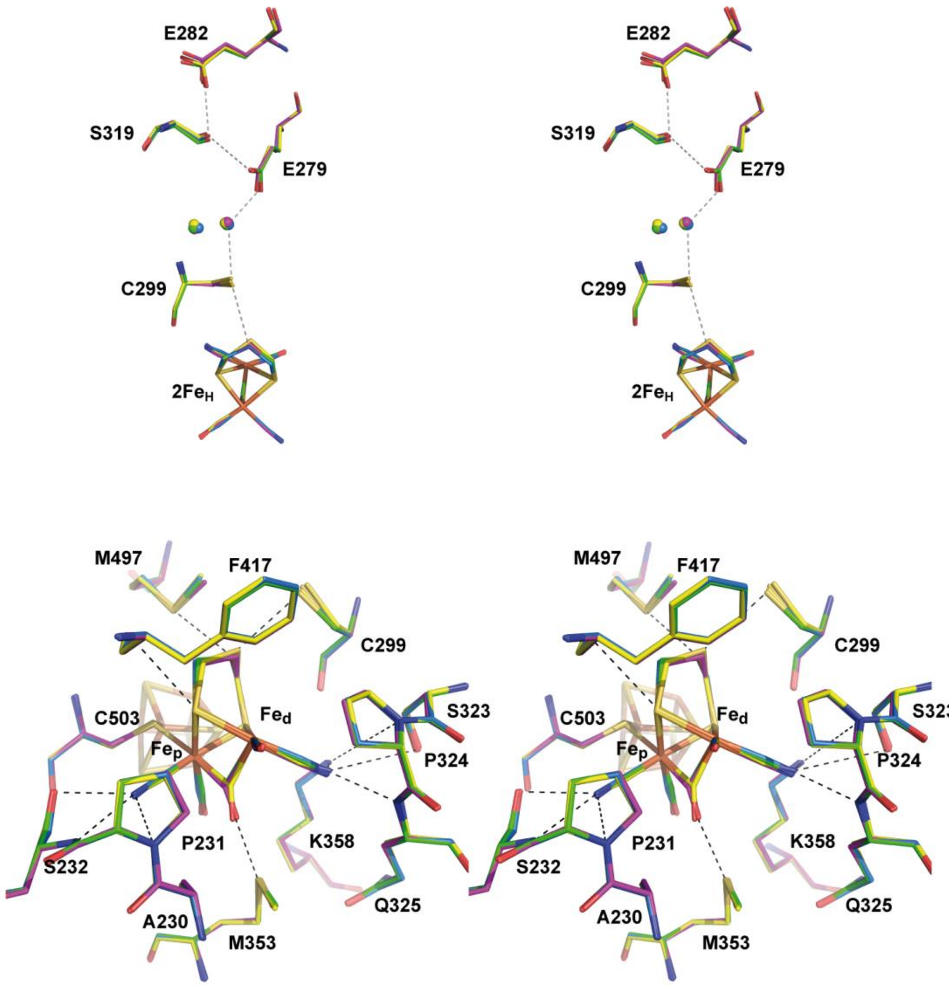

Figure S5: Stereo view of Figure 5: Comparison of the catalytically important amino acids in CpI derivatives. Stick models of the potential proton transfer pathway (A) and the environment of the $2Fe_H$-subcluster (B) of CpI^{ADT} (carbon atoms in marine) superposed to stick models of CpI^{PDT} (magenta), CpI^{ODT} (green) and CpI^{SDT} (yellow). Dashed lines indicate potential proton transfer interactions or potential interactions of $2Fe_H$ with the protein as listed in table S4. Numbering of amino acids as in the structure of native CpI.

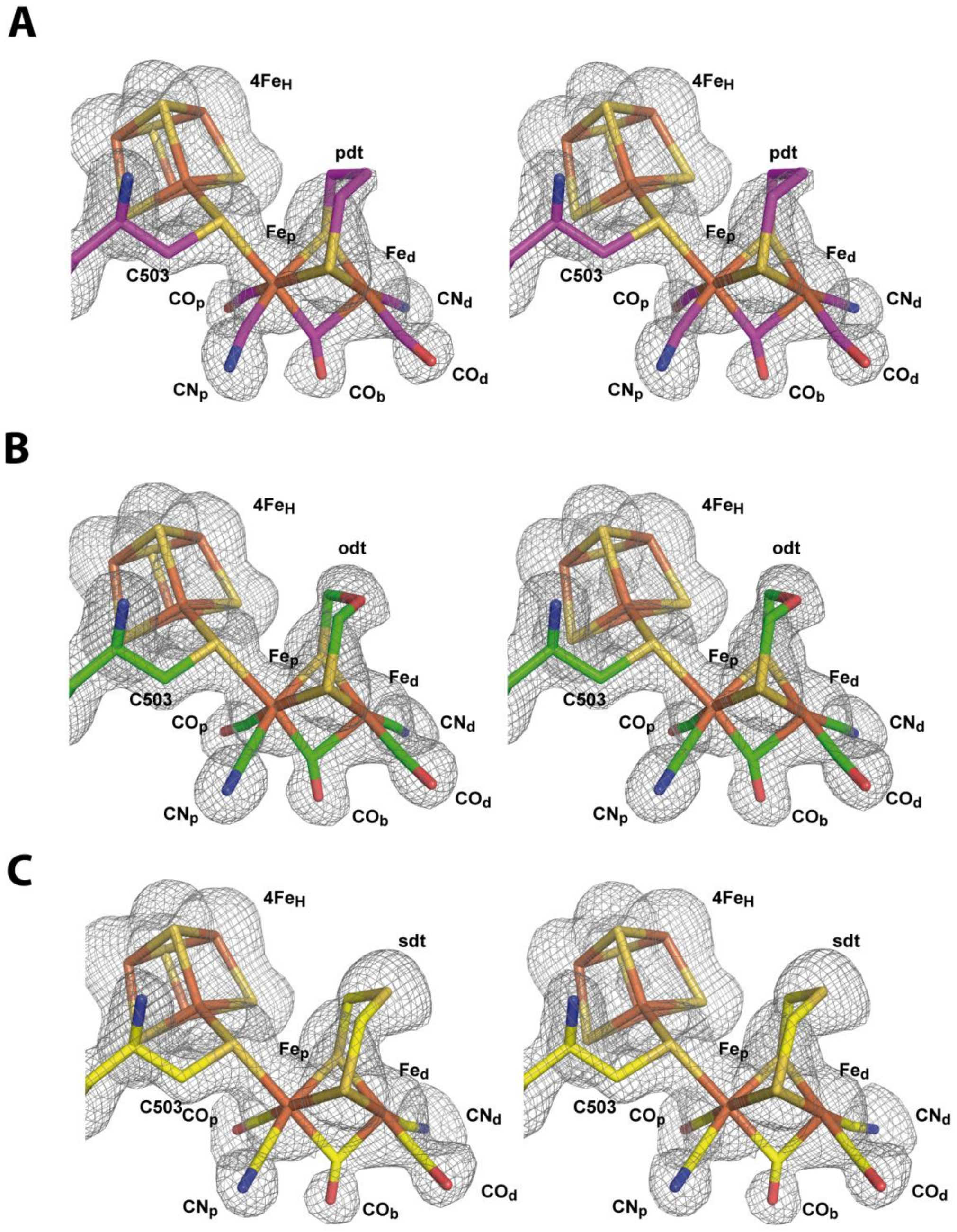

Figure S6: Stereo view of Figure 6: Models of the H-cluster of non-native CpI derivatives. Stick models of (A) CpI^{PDT} (carbon atoms in magenta), (B) CpI^{ODT} (carbon atoms in green) and (C) CpI^{SDT} (carbon atoms in yellow) with F_o-F_c simulated annealing omit maps contoured at 3.5σ.

References

Lambertz, C.; Chernev, P.; Klingan, K.; Leidel, N.; Sigfridsson, K. G. V.; Happe, T.; Haumann, M. Electronic and Molecular Structures of the Active-Site H-Cluster in [FeFe]-Hydrogenase Determined by Site-Selective X-Ray Spectroscopy and Quantum Chemical Calculations. *Chem. Sci.* **2014**, *5* (3), 1187–1203.

Li, H.; Rauchfuss, T. B. Iron Carbonyl Sulfides, Formaldehyde, and Amines Condense To Give the Proposed Azadithiolate Cofactor of the Fe-Only Hydrogenases. *J. Am. Chem. Soc.* **2002**, *124* (5), 726–727.

Lovell, S. C.; Davis, I. W.; Arendall, W. B.; de Bakker, P. I. W.; Word, J. M.; Prisant, M. G.; Richardson, J. S.; Richardson, D. C. Structure Validation by Cα Geometry: Φ,ψ and Cβ Deviation. *Proteins Struct. Funct. Bioinforma.* **2003**, *50* (3), 437–450.

Mulder, D. W.; Boyd, E. S.; Sarma, R.; Lange, R. K.; Endrizzi, J. A.; Broderick, J. B.; Peters, J. W. Stepwise [FeFe]-Hydrogenase H-Cluster Assembly Revealed in the Structure of HydA ΔEFG. *Nature* **2010**, *465* (7295), 248–251.

Pandey, A. S.; Harris, T. V.; Giles, L. J.; Peters, J. W.; Szilagyi, R. K. Dithiomethylether as a Ligand in the Hydrogenase H-Cluster. *J. Am. Chem. Soc.* **2008**, *130* (13), 4533–4540.

Schmidt, M.; Contakes, S. M.; Rauchfuss, T. B. First Generation Analogues of the Binuclear Site in the Fe-Only Hydrogenases: Fe2(μ-SR)2(CO)4(CN)22-. *J. Am. Chem. Soc.* **1999**, *121* (41), 9736–9737.

Song, L.-C.; Yang, Z.-Y.; Bian, H.-Z.; Hu, Q.-M. Novel Single and Double Diiron Oxadithiolates as Models for the Active Site of [Fe]-Only Hydrogenases. *Organometallics* **2004**, *23* (13), 3082–3084.

Song, L.-C.; Yang, Z.-Y.; Hua, Y.-J.; Wang, H.-T.; Liu, Y.; Hu, Q.-M. Diiron Thiadithiolates as Active Site Models for the Iron-Only Hydrogenases: Synthesis, Structures, and Catalytic H2 Production. *Organometallics* **2007**, *26* (8), 2106–2110.

Winkler, M.; Esselborn, J.; Happe, T. Molecular Basis of [FeFe]-Hydrogenase Function: An Insight into the Complex Interplay between Protein and Catalytic Cofactor. *Biochim. Biophys. Acta BBA - Bioenerg.* **2013**, *1827* (8–9), 974–985.
